# Percutaneous transforaminal endoscopic surgery combined with mini-incision OLIF and anterolateral screws rod fixation vs. MIS-TLIF for surgical treatment of single-level lumbar spondylolisthesis

**DOI:** 10.3389/fsurg.2022.1049448

**Published:** 2023-01-06

**Authors:** Tianyao Zhou, Wenshuai Fan, Yutong Gu, Wu Che, Liang Zhang, Yichao Wang

**Affiliations:** ^1^ Department of Orthopaedic Surgery, Zhongshan Hospital Fudan University, Shanghai, China; ^2^ Department of Orthopaedic Surgery, Shanghai Southwest Spine Surgery Center, Shanghai, China; ^3^ Department of Orthopaedics, Ruijin Hospital, Shanghai Jiao Tong University School of Medicine, Shanghai, China

**Keywords:** lumbar spine spondylolisthesis, percutaneous transforaminal endoscopic surgery, oblique lumbar interbody fusion, transforaminal lumbar interbody fusion, screws rod fixation

## Abstract

**Objective:**

Oblique lumbar interbody fusion (OLIF) has been used to treat lumbar spine spondylolisthesis. However, it usually needs posterior pedicle screws fixation for biomechanical stability and possible posterior direct decompression for relieving neurologic symptoms. We use percutaneous transforaminal endoscopic surgery (PTES) combined with mini-incision OLIF and anterolateral screws rod fixation for surgical treatment of lumbar spondylolisthesis. The purpose of study is to evaluate the feasibility, efficacy, and safety of this method compared with minimally invasive surgery-transforaminal lumbar interbody fusion (MIS-TLIF).

**Methods:**

From July 2016 to May 2018, 65 patients of lumbar spondylolisthesis (L2–4) with neurologic symptoms were treated using PTES combined with mini-incision OLIF and anterolateral screws rod fixation (31 cases, group A) or MIS-TLIF (34 cases, group B) in this study. Operative duration, blood loss, incision length, fluoroscopy frequency, and hospital stay are compared. Preoperative and postoperative visual analog scale (VAS) pain scores of back and legs, Oswestry disability index (ODI), intervertebral space height, lumbar lordotic angle, operative segmental lordotic angle, and complications are recorded. The fusion status is assessed according to Bridwell's fusion grades.

**Results:**

The VAS score of back and leg pain and ODI significantly dropped after surgery in both groups (*p* < 0.001). There was no statistical difference of back and leg VAS score and ODI between two groups except that back VAS scores in group A were significantly lower than that of group B immediately after surgery (*p* = 0.000). Group A had significantly more intervertebral space height and operative segmental lordotic angle than group B postoperatively (*p* = 0.022, *p* = 0.002). Twenty-three segments (74.2%) were grade I and 8 segments (25.8%) were grade II in group A; 20 segments (58.8%) were grade I and 14 segments (41.2%) were grade II in group B at a 2-year follow-up (*p* = 0.194). No difference was observed in the complication rate between the two groups (6.5% vs. 5.9%, *p* = 0.924).

**Conclusion:**

The long-term clinical efficacy and complication rates of both groups are comparable. PTES combined with mini-incision OLIF and anterolateral screws rod fixation is a good choice of minimally invasive surgery for lumbar spondylolisthesis, which hardly destroys the paraspinal muscles and bone structures.

## Introduction

Lumbar degeneration and spondylolysis are the main reasons for lumbar spine spondylolisthesis to happen (1, 2). When conservative treatment fails, lumbar interbody fusion and neurologic decompression become the standard surgical treatment. Lumbar interbody fusion surgery was initially invented to treat spinal tuberculosis (3, 4). In 1948, Lane and Moore (5) first applied lumbar interbody fusion for the treatment of lumbar degenerative diseases and obtained encouraging result of relieving symptoms. Since then, the indication of lumbar interbody fusion has been widened. Nowadays, lumbar interbody fusion is applied to patients with lumbar disc herniation, spondylolisthesis, pseudoarthrosis, and spinal deformities (6).

In 1997, Mayer (7) reported an anterior to psoas surgical trajectory for lumbar interbody fusion. In 2012, Silvestre et al. (8) named the approach oblique lumbar interbody fusion (OLIF). OLIF has been used to treat lumbar spine spondylolisthesis, which has some advantages including less damage to paraspinal muscles and bone structures, less blood loss, and faster recovery. Compared with posterior lumbar interbody fusion (PLIF) or transforaminal lumbar interbody fusion (TLIF), OLIF uses a bigger cage to achieve higher fusion rate by getting more touch surface between endplate of vertebra and cage and implanting more graft bone (9, 10). In addition, bigger cage has better distraction ability of intervertebral space helpful for restoration of intervertebral space height and lumbar lordotic angle and reduction of spondylolisthesis.

However, posterior instrumentation is usually needed to enhance the biomechanical stability of OLIF (11). Sometimes there is no improvement of neurologic symptoms after surgery due to indirect and inadequate decompression of OLIF (10, 12). Further posterior surgery sharply reduces the advantages of OLIF resulting from longer operative time under general anesthesia and more invasiveness (13). In this study, OLIF and anterolateral screws rod fixation in the same mini-incision was combined with percutaneous transforaminal endoscopic surgery (PTES) (14, 15) under local anesthesia for the treatment of single level lumbar spine spondylolisthesis in order to obtain direct decompression, good reduction, rigid fixation, high fusion rate, and protect the paraspinal muscles and bone structures as much as possible. The purpose of study is to evaluate the feasibility, efficacy, and safety of this combination compared with minimally invasive surgery-transforaminal lumbar interbody fusion (MIS-TLIF).

## Materials and methods

### Patients

The clinical study proposal was approved by the Medical Ethics Committee of Zhongshan Hospital, Fudan University. Informed consent was obtained from all individual participants for using their imaging data and questionnaire scores. From July 2016 to May 2018, 65 patients of single lumbar spondylolisthesis from L1 to L4 with neurologic symptoms were treated using PTES combined with mini-incision OLIF and anterolateral screws rod fixation (31 cases, group A) or MIS-TLIF (34 cases, group B) in this study.

**Figure 1:**
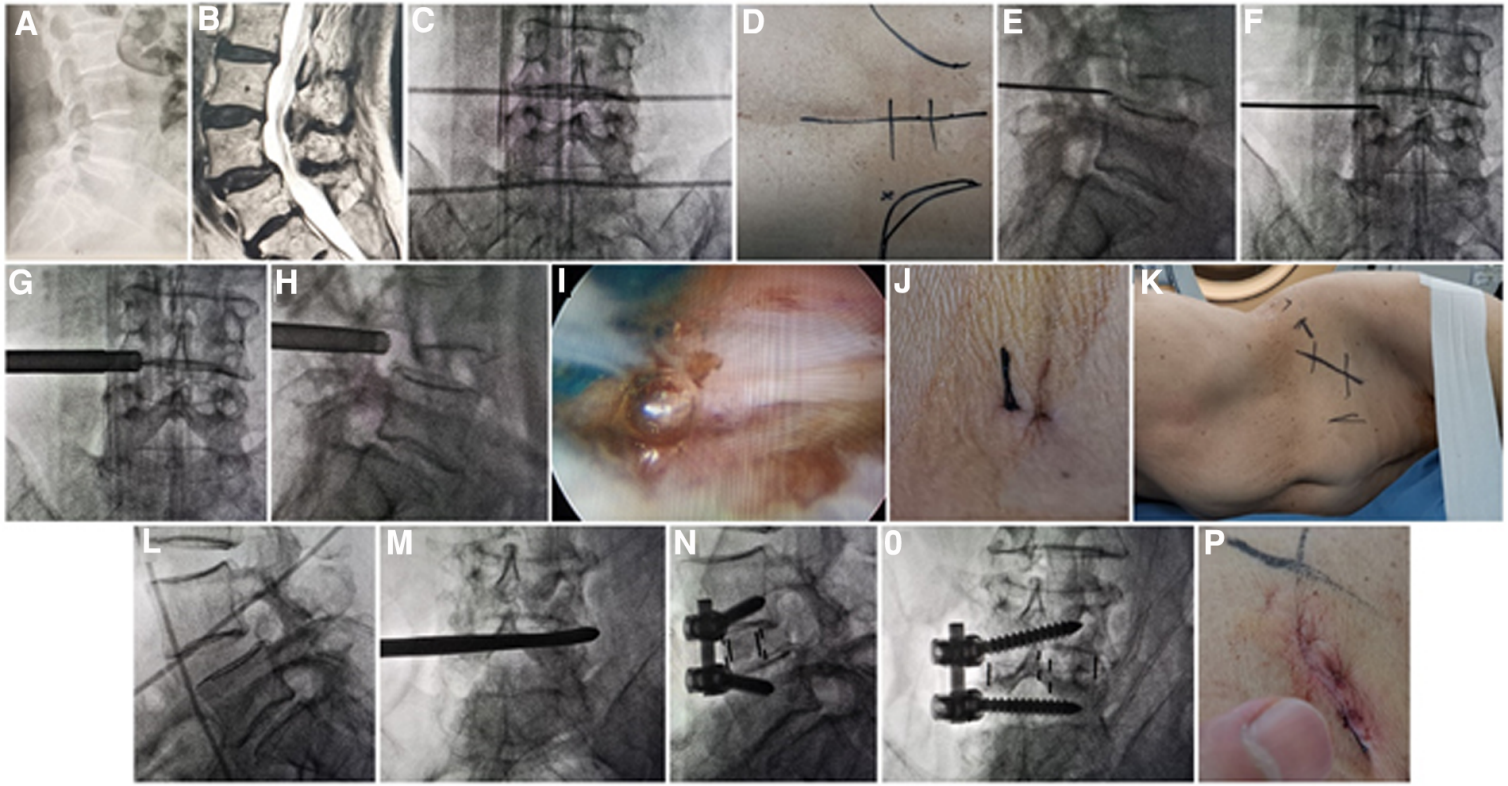
(**A**) X-ray and (**B**) MRI showed L4 Spondylolisthesis (I°) caused by degeneration in a 76-year-old female patient. Metal rods were placed transversely across the center of the target disc on (**C**) posteroanterior C-arm view to draw transverse lines. (**D**) The aimed reference point of puncture at surface was identified by the intersection of transverse line and longitudinal midline, and the entrance point of puncture (Gu's point) was located at the corner of flat back turning to lateral side. During puncture, once resistance disappeared, the C-arm view was taken to ensure that the tip of puncture needle was in the intracanal area close to the posterior wall of disc on (**E**) lateral x-ray and near the lateral border of pedicle on (**F**) posteroanterior x-ray. During press-down enlargement of foramen, when resistance disappeared, the tip of reamer should exceed the medial border of pedicle on (**G**) posteroanterior C-arm view and reach close to the posterior wall target disc on (**H**) lateral C-arm view. Under (**I**) endoscopic view, the compressed nerve root was freed after the hypertrophic ligamentum flavum and herniated disc were removed. (**J**) The stab incision of about 8 mm for PTES was closed. (**K**) Patient was placed into right decubitus position for mini-incision OLIF and anterolateral screws rod fixation. The anterior line of L4/5 intervertebral space was positioned using (**L**) C-arm view. The spatula was inserted into L4/5 intervertebral space to cut the contralateral fibrous annulus, which was confirmed by (**M**) x-ray image. After the OLIF cage was placed into disc space parallel to the endplate and anterolateral screws rod fixation was performed, (**N**) lateral and (**O**) posteroanterior C-arm view confirmed good position of internal instruments. (**P**) The mini-incision of OLIF was sutured finally. PTES, percutaneous transforaminal endoscopic surgery; OLIF, oblique lumbar interbody fusion.

**Figure 2:**
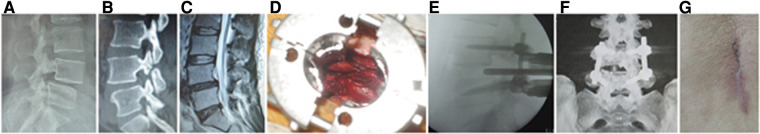
(**A**) X-ray, (**B**) CT, and (**C**) MRI showed L4 spondylolisthesis (I°) caused by spondylolysis in a 44-year-old female patient. (**D**) An expandable tubular retractor was placed to undertake unilateral complete facetectomy and hemilaminectomy and expose dural sac nerve root. The cage was inserted into L4/5 intervertebral space on (**E**) lateral C-arm view and (**F**) 3D CT reconstruction showed the screws, cage, and neurologic decompression in MIS-TLIF. (**G**) The incision of MIS-TLIF was closed. MIS-TLIF, minimally invasive surgery-transforaminal lumbar interbody fusion.

The inclusion criteria were as follows: (1) low back pain and unilateral or bilateral asymmetry leg pain or bilateral symmetry legs pain when rest, or intermittent claudication with no symptom of legs when rest and symmetry pain, numbness, discomfort, or tiredness of both legs after walking 50 m–100 m, unable to walk, relieved after rest; (2) image data of x-ray, MRI, and CT show single lumbar spondylolisthesis [Meyerding (16) I° or II°] from L2 to L4 (Figures 1A,B, 2A–C), which is consistent with neurologic symptoms; (3) outcome is poor after at least 3 months of regular conservative treatment, and symptoms severely affect work and daily life; (4) the systemic status is good, basic medical diseases such as heart disease, hypertension or diabetes are under control, and the mental state is normal; (5) with complete data and perioperative records, as well as radiographic follow-up data.

The exclusionary criteria were the presence of more than two-level lumbar spondylolisthesis, previous lumbar interbody fusion, spinal tumor, spinal infection, other medical conditions making the patient intolerant to operation, inability to give informed consent, and a likelihood of noncompliance with follow-up.

### Preoperative and postoperative imaging

All patients are evaluated before the procedure by CT and MRI imaging to determine lumbar spondylolisthesis, disc herniation, lateral recess stenosis, intervertebral foramen stenosis, or central spinal canal stenosis. Posteroanterior and lateral radiographs are obtained to assess the slip degree of vertebral body according to the Meyerding Classification System of Spondylolisthesis (16). Intervertebral space height (17), lumbar lordotic angle, and operative segmental lordotic angle are measured on lumbar spine x-rays at preoperative, postoperative, and 2-year follow-up. The intervertebral space height is the average of anterior and posterior spaces between two adjacent vertebrae on the lateral x-ray; the lumbar lordotic angle is the angle between the upper endplate of first lumbar vertebra and the upper endplate of sacrum; the operative segmental lordotic angle is the angle between the upper endplate of upper vertebra and the lower endplate of lower vertebra in the surgical segment. A loss of at least 2 mm of intervertebral space height is generally considered cage subsidence on x-ray (17). The fusion status is assessed according to Bridwell's fusion grades on CT (18). After the treatment, MRI images are obtained to assess neurologic decompression or exclude dural cyst, myelomeningocele, dural tears or spinal fluid leaks, and reherniation.

### Surgical procedure

All the surgeries were undertaken by the same senior surgeon. C-arm was used for intraoperative fluoroscopic imaging.

#### Group A: PTES + mini-incision OLIF and anterolateral screws rod fixation

The patient is in a prone position on a radiolucent table for PTES under local anesthesia with conscious sedation. The intersection of posterior midline and the transverse line of surface marking of target disc is the aiming reference point of puncture (Figures 1C,D). The entrance point of puncture locates at the corner of flat back turning to lateral side at the height of target disc, or cranially or slightly caudally. This entrance point, named “Gu's point,” is easy to determine without the fluoroscopy regardless of different age, gender, and body size (14, 15) (Figure 1D). An 18-gauge puncture needle is inserted anteromedially at an angle of about 45°(25°–85°) to horizontal plane. After the success of puncture (Figures 1E,F) and dilating the puncture tract stepwise, an 8.8-mm diameter cannula with one-side opening is inserted over the guiding rod and docked at the superior facet. Then the cannula is pressed down to decrease the inclination angle, and a 7.5-mm diameter hand reamer is introduced through the cannula to remove the ventral bone of articular process to enlarge the foramen. When resistance disappears, the tip of the reamer should exceed the medial border of pedicle on posteroanterior view and reach close to the posterior wall target disc on lateral view. (Figures 1G,H) This procedure is named “press-down enlargement of foramen” (14, 15). For lumbar central spinal canal stenosis, it is repeated to remove more ventral bone of articular process. The 7.5-mm diameter working cannula is inserted over the guiding rod, and the hypertrophic ligamentum flavum and herniated disc are removed to enlarge the lateral recess, and the compressed ipsilateral nerve root, even the contralateral nerve root, are exposed for unilateral or bilateral decompression under the endoscope (Figure 1I). The dorsal dural sac is exposed to enlarge the central spinal canal. The patients can feel the symptomatic legs obviously relaxed after the culprit segment is treated, and the incision is closed (Figure 1J).

Then the patients are placed into a right lateral decubitus position under controlled general anesthesia with trachea cannula to undergo mini-incision OLIF and anterolateral screws rod fixation for spondylolisthesis segment (Figure 1K). The preoperative C-arm is used to position the surface mark of anterior edge of target intervertebral space (Figure 1L), and the mini-incision is located along the anterior edge of intervertebral space or iliac crest. After the skin and subcutaneous tissues are incised, the external oblique, internal oblique, and transverse abdominal muscles are bluntly separated in turn to enter the retroperitoneal space and expose the anterior border of psoas major muscle with two narrow long retractors. After fluoroscopic projection for confirming the surgical segment, the intervertebral fibrous annulus is opened from the lateral side along the anterior border of psoas major muscle, and the spatula is inserted into intervertebral space to cut the contralateral fibrous annulus (Figure 1M). The intervertebral tissue is removed, and upper and lower cartilage endplates are adequately scraped off, taking care to avoid damaging the bony endplates during the operation. After trial molding, the OLIF cage (Medtronic, Inc., Minneapolis, MN, United States) of appropriate size is filled with allograft bone and autograft obtained during PTES, and placed into disc space parallel to the endplate. Through the same approach, two pedicle screws (Medtronic, Inc., Minneapolis, MN, United States) are inserted into adjacent vertebrae from the anterolateral side close to the endplate. Finally, after the fluoroscopic view of cage and screws is satisfactory, the rod is fixed over the screws (Figures 1N,O), and the surgical incision is closed layer by layer with a thin drain tube (Figure 1P).

#### Group B: MIS-TLIF

The patients are in the prone position on a radiolucent operating table under general anesthesia. After localization with fluoroscopy, bilateral paraspinal muscle-splitting approaches through a 3.5-cm midline incision are performed to expose facet joints and transverse processes of the upper and lower vertebrae. The pedicle screws (DePuy, Inc., Warsaw, IN, United States) are placed at the junction between the lateral facet wall and the middle transverse process. After sequential dilation, an expandable tubular retractor (DePuy, Inc., Warsaw, IN, United States) is placed, and a unilateral complete facetectomy and hemilaminectomy are undertaken to expose the dura and nerve root involved (Figure 2D). Then, the disc material and cartilaginous endplate are removed, and sufficient autologous bone graft that was obtained is packed in the disc space before a cage (DePuy, Inc., Warsaw, IN, United States) filled with autograft bone obliquely inserted (Figure 2E). After removing the expandable retractor, two rods are fixed with pedicle screws (Figure 2F). The suction drain is placed, and the wound is closed in layers (Figure 2G). If there is dural tear, the lumbodorsal fascia must be sutured very tightly to prevent cerebrospinal fluid leakage from surgical incision.

Operative duration, blood loss, incision length, fluoroscopy frequency, and hospital stay are recorded. Patients could walk with a flexible brace after the drain tube is removed when the drainage fluid is less than 20 ml/24 h. If cerebrospinal fluid leakage from drain occurs, the drainage tube should be removed 7 days after surgery when the wound heals. After leaving hospital, patients are encouraged to return to daily life and followed up regularly.

### Clinical follow-up

Back and leg pain are evaluated using the 10-point visual analog scale (VAS) preoperatively, immediately, 1, 2, 3, and 6 months, 1, and 2 years after surgery. The clinical outcomes are evaluated with Oswestry Disability Index (ODI) at a 2-year follow-up. During the follow-up, all complications are recorded including iatrogenic nerve damage, vascular injuries, infection, wound healing, thrombosis, or recurrence.

### Statistical analysis

SPSS 25 software (SPSS Inc., Chicago, IL, United States) was used to perform statistical analysis, and a value of less than 0.05 was considered statistical significance. Normal distributed continuous variables such as age, operative duration, incision length, follow-up, ODI, intervertebral space height, lumbar lordotic angle, and operative segmental lordotic angle are presented as mean ± standard deviation (SD); discrete, rating, and continuous variables, which are not normally distributed, are presented as median (maximum–minimum) including fluoroscopy frequency, blood loss, drainage removal, hospital stay, and VAS; categorical variables such as gender, inducement, lumbar level, and complication rate are expressed as frequency or percentage. Student’s *t*-test is used for intergroup analysis of normal distributed continuous variables. The Mann–Whitney *U* test is used for intergroup analysis of discrete, rating, and continuous variables, which are not normally distributed. The *χ*^2^ test is used for intergroup analysis of categorical variables. The one-way ANOVA followed by the Tukey *post hoc* analysis is performed for intragroup comparison of VAS, intervertebral space height, lumbar lordotic angle, and operative segmental lordotic angle at different time points. The ODI score before the treatment and 2 years after surgery are compared using Student‘s *t*-test.

## Results

Clinical data are summarized in Table 1. In group A, 31 patients were treated with PTES combined with mini-incision OLIF and anterolateral screws rod fixation. In group B, 34 patients obtained MIS-TLIF. There were no significant differences in age, gender, inducement, and lumbar level between the two groups. The patients in group A had significantly less operative duration under general anesthesia (75 ± 13 min vs. 104 ± 18 min, *p* = 0.000), less blood loss (30/15–110 ml vs. 80/50–310 ml, *p* = 0.000), earlier drainage removal (2/1–3 days vs. 4/3–7 days, *p* = 0.000), and shorter hospital stay (4/3–5 days vs. 7/6–10 days, *p* = 0.000) than group B did. There was a significant difference in the incision length (39 ± 3 mm vs. 41 ± 3 mm, *p* = 0.006) and no statistical difference in fluoroscopy frequency (7/5–10 times vs. 7/6–11 times, *p* = 0.176) between mini-incision OLIF of group A and MIS-TLIF of group B, but another incisions of 8 ± 1 mm, fluoroscopy of 6/5–8 times, and operative duration under local anesthesia of 50 ± 8 min were needed for PTES besides OLIF in group A.

**Table 1:** Comparison of clinical data between group A and B.

	Group A	Group B	*p* value
Age (years)	60 ± 8	61 ± 7	0.527
Gender			
Female	12	21	0.969
Male	19	13	
Inducement			
Degeneration	14	12	0.417
Spondylolysis	17	22	
Level			
L3	3	4	0.786
L4	28	30	
Operative duration (min)	OLIF 75 ± 13	104 ± 18	0.000^a^
		PTES 50 ± 8	
Fluoroscopy (times)	OLIF 7/5–10	7/6–11	0.176^a^
		PTES 6/5–8	
Incision length (mm)	OLIF 39 ± 3	41 ± 3	0.006^a^
		PTES 8 ± 1	
Blood loss (ml)	30/15–110	80/50–310	0.000
Hospital stay (days)	4/3–5	7/6–10	0.000
Drainage removal (days)	2/1–3	4/3–7	0.000
Follow-up (months)	32 ± 3	32 ± 4	0.632

OLIF, oblique lumbar interbody fusion; MIS-TLIF, minimally invasive surgery-transforaminal lumbar interbody fusion.

^a^ Comparison between OLIF and MIS-TLIF.

The preoperative back VAS scores were 6 (4–10) in two groups, which obviously decreased to 1 (0–3) immediately after surgery and 0 (0–2) at 2-year follow-up in group A, whereas those in group B were significantly decreased to 3 (2–5) immediately after surgery and 1 (0–2) at 2-year follow-up. The leg VAS score significantly dropped from 9 (7–10) of group A and 8 (7–10) of group B preoperatively to 1 (0–3) immediately after surgery and 0 (0–2) at 2-year follow-up in both groups, respectively. There was no statistical difference of leg VAS scores in two groups after surgery. However, back VAS scores in group A was significantly lower than that of group B immediately after surgery (*p* = 0.000) (Tables 2 and 3). The preoperative ODI was 66.9% ± 8.8% in group A and 67.2% ± 9.0% in group B, which significantly decreased to 15.0% ± 5.3% in group A and 15.6% ± 4.7% in group B, and no statistical difference of ODI was found between the two groups (*p* = 0.643) (Table 4).

**Table 2:** VAS pain assessments of back between two groups.

Group	Preoperative	Postoperative	1 month	2 months	3 months	6 months	1 year	2 years
A	6 (4–10)	1 (0–3)	0 (0–2)	0 (0–2)	0 (0–2)	0 (0–2)	0 (0–2)	0 (0–2)
B	6 (4–10)	3 (2–5)	1 (0–2)	1 (0–2)	1 (0–2)	1 (0–2)	1 (0–2)	1 (0–2)
*p* value	0.536	0.000	0.001	0.197	0.197	0.133	0.133	0.133

VAS, visual analog scale.

**Table 3:** VAS pain assessments of legs between two groups.

Group	Preoperative	Postoperative	1 month	2 months	3 months	6 months	1 year	2 years
A	9 (7–10)	1 (0–3)	0 (0–2)	0 (0–2)	0 (0–2)	0 (0–2)	0 (0–2)	0 (0–2)
B	8 (7–10)	1 (0–3)	0 (0–2)	0 (0–2)	0 (0–2)	0 (0–2)	0 (0–2)	0 (0–2)
*p* value	0.468	0.760	0.628	0.728	0.927	0.721	0.721	0.721

VAS, visual analog scale.

**Table 4:** ODI of two groups (%).

Group	Preoperative	2 years
A	66.9 ± 8.8	15.0 ± 5.3
B	67.2 ± 9.0	15.6 ± 4.7
*p* value	0.911	0.643

ODI, Oswestry disability index.

**Figure 3:**
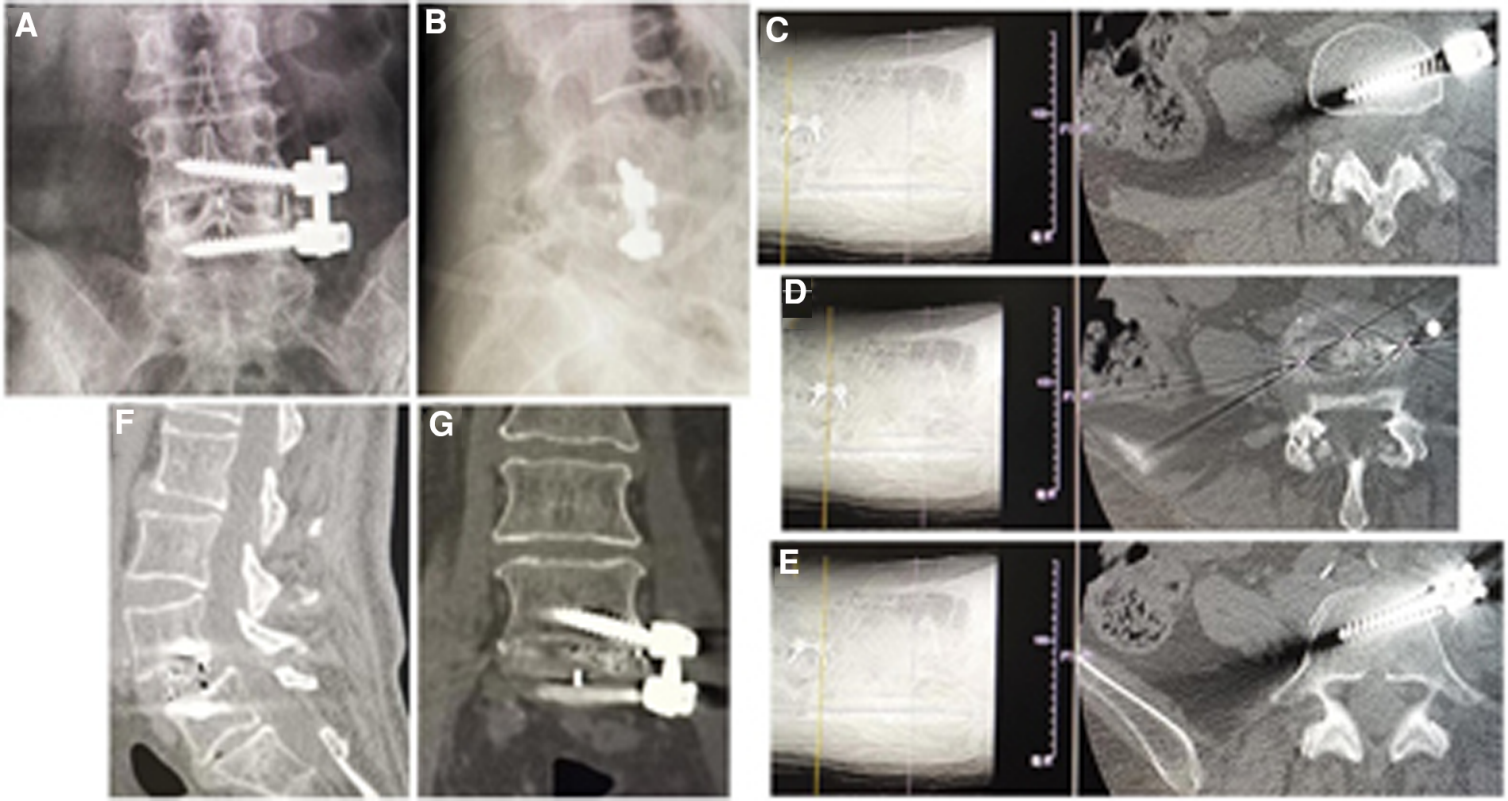
(**A**) Posteroanterior and (**B**) lateral x-ray image, and (**C–E**) axial CT images showed good position of cage and screws after operation. Fusion grade at 2-year follow-up was grade I on (**F**) sagittal and (**G**) coronal CT image.

**Figure 4:**
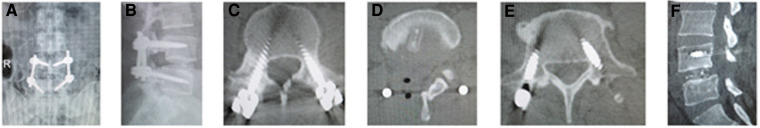
(**A**) Posteroanterior and (**B**) lateral x-ray image, and (**C–E**) axial CT image showed good position of cage and screws after operation. Fusion grade at 2-year follow-up was grade II on (**F**) sagittal CT image.

The postoperative x-ray and CT scans demonstrated good position of cage and screws (Figures 3A–E and 4A–E). The intervertebral space height, lumbar lordotic angle, and operative segmental lordotic angle significantly improved postoperatively in two groups, and no significant difference change was observed at 2-year follow-up. However, group A showed significantly more intervertebral space height and operative segmental lordotic angle than those of group B postoperatively (*p* = 0.022, *p* = 0.002) (Tables 5–7). At 2-year follow-up, fusion grades based on the Bridwell grading system were grade I for 23 segments (74.2%) (Figures 3F,G) and grade II for 8 segments (25.8%) in group A, and grade I for 20 segments (58.8%) and grade II for 14 segments (41.2%) (Figure 4F) in group B (*p* = 0.194).

**Table 5:** Intervertebral space height of two groups (mm).

Group	Preoperative	Postoperative	2 years
A	9.5 ± 1.7	12.9 ±1.5	12.3 ± 1.5
B	9.6 ± 2.0	12.0 ± 1.7	11.1 ± 1.7
*p* value	0.840	0.022	0.006

**Table 6:** Lumbar lordotic angle of two groups (°).

Group	Preoperative	Postoperative	2 years
A	40.1 ± 9.7	48.5 ± 8.5	45.8 ± 8.7
B	40.0 ± 9.1	46.2 ±8.9	43.5 ± 8.7
*p* value	0.974	0.302	0.302

**Table 7:** Operative segmental lordotic angle of two groups (°).

Group	Preoperative	Postoperative	2 years
A	15.8 ± 2.9	20.2 ± 2.8	18.3 ± 2.8
B	15.4 ± 3.4	17.3 ± 3.3	16.9 ± 3.3
*p* value	0.679	0.002	0.060

There were two cases of hip flexion pain and weakness, which was relieved during 1 week after surgery in group A. In group B, two patients encountered dural tear and cerebrospinal fluid leakage from drain without neurologic symptoms. Their wound healed after the drainage tube was removed 7 days postoperatively. No other complications such as wound infection, permanent nerve injury, ruptured large vessels, hardware failure, and cage subsidence were observed. There was no difference in complication rate between two groups (6.5% vs. 5.9%, *p* = 0.924).

## Discussion

In OLIF, indirect neurologic decompression is achieved by placing the big cage into disc space to increase disc height, which can tighten the posterior longitudinal ligament, enlarge the cross-sectional area (CSA) of spinal canal, and intervertebral foramen to alleviate the pressure on neurologic elements, as Lin et al. (19) indicated in their study. In the radiographic study of Limthongkul et al. (20), the CSA of thecal sac increased from 93.1 mm ± 43.0 mm to 127.3 mm ± 52.5 mm (50.8%; *p* value < 0.00625) after OLIF. Beng et al. (21) divided the patients into three groups based on their preoperative lumbar lordosis: group A, <0°; group B, 0°–20°; and group C, >20°. The mean CSA enlargement ratios were 27.5%, 32.1%, and 60.4% in groups A, B, and C, respectively. To some extent, this can relieve the patient's symptoms (22). However, sometimes OLIF alone had no improvement of neurologic symptoms because of the inadequate decompression (10, 23). The study of Li et al. (23) showed that the overall posterior direct decompression rate after OLIF was 29.97%, and extreme severe lumbar central canal stenosis is the greatest determinant to perform the second-stage posterior direct decompression procedure after OLIF. Lim et al. (24) and Yingsakmongkol et al. (25) found that persistent pain despite resting in a supine position suggests the presence of severe spinal canal stenosis with significant static nerve compression that would only be sufficiently relieved with a direct decompression after extreme lateral interbody fusion. The segment is too rigid to be restored, which indicates that it is difficult to obtain a greater postoperative disc height and more indirect decompression effect (24). Some studies showed that bony lateral recess stenosis is an independent predictor for failure to achieve adequate spinal decompression *via* indirect decompression with lateral lumbar interbody fusion (25–27). Additionally, the free herniated disc migrating into spinal canal or head, or tail of the involved segment could not be treated by indirect decompression of OLIF. Further posterior direct decompression after OLIF needs another general anesthesia and increases the aggressiveness and medical expenses.

Most patients with lumbar spondylolisthesis have symptoms of nerve root compression, such as one-leg pain or numbness, asymmetric pain, or numbness in both legs or symmetric pain of both legs when rest, which results from lumbar disc herniation, lateral recess stenosis, or intervertebral foramen stenosis. Few patients have both-leg symmetric pain, numbness, discomfort, and/or tiredness occurring after walking for 50–100 m, which could be relieved after a few minutes of rest. This is intermittent claudication of both legs resulting from *cauda equina* compression and should be diagnosed as lumbar central spinal canal stenosis. In 2017, we first introduced our PTES (14) with reduced steps, simple orientation and easy puncture, which can significantly decrease the times of fluoroscopy projection and shorten the operation time. We used PTES to successfully treat lumbar degenerative diseases with neurologic symptoms including lumbar spondylolisthesis (14, 15). During the procedure, we performed press-down enlargement of foramen to saw off the ventral bone of articular process. In addition, the hypertrophic *ligamentum flavum* and the protruding *nucleus pulposus* were removed to expand the lateral recess and reduce the pressure of nerve root. The ipsilateral and contralateral nerve roots can be exposed, and the bilateral nerve roots can be decompressed from one side through a small incision. When there is lumbar central spinal canal stenosis, press-down enlargement of foramen was repeated to remove more ventral bone of articular process and expose dural sac for almost 180° enlargement of central spinal canal. In this study, we performed PTES under local anesthesia for direct decompression before OLIF, which could guarantee the relief of neurologic symptoms and avoid another entrance into operation room. If the indirect decompression of OLIF has no effect, the reoperation, even PTES under local anesthesia, could put more psychological pressure on the patients and the surgeons, especially in China due to the complicated doctor–patient relationship. The results confirm that the VAS score of leg pain and ODI significantly dropped after surgery in both groups (*p* < 0.001). The clinical efficacy of PTES combined with mini-incision OLIF and anterolateral screws rod fixation is similar to that of MIS-TLIF.

According to Soriano-Baron et al. (11), OLIF alone maintained axial compressive stiffness when comparing with the intact condition, and the surgeon should consider the biomechanical and patient-specific factors for selecting the appropriate supplement fixation technique for any interbody spacers. The study of Guo et al. (28) showed that OLIF alone could not provide sufficient stability and need additional fixation. Bilateral pedicle screws (BPS) fixation has the most rigid structure but requires paraspinal muscle dissection and retraction during instrumentation, has neurologic risk, vascular injury, and increased operative time. Unilateral pedicle screw (UPS) fixation involves less damage to the paravertebral muscles, less perioperative bleeding, and low instrument expense, but it offers significantly less stability than the BPS. Compared with BPS and UPS, lateral rod-screw fixation may be appropriate for patients with good bone quality, normal body mass index, and nonspondylolisthetic lumbar fusion (28). The additional posterior surgery of pedicle screws fixation enlarges the aggressiveness, prolongs the time of general anesthesia, or furthermore requires another general anesthesia. In this study, we inserted the pedicle screws into vertebral bodies and fixed the rod over screws from the anterolateral side after the placement of cage in the same mini-incision of OLIF. The results of our study showed that all patients of PTES combined with mini-incision OLIF and anterolateral screws rod fixation got fusion at 2-year follow-up, and no failure of instruments was observed. These confirmed that anterolateral screws rod fixation and OLIF can supply good biomechanical property for intervertebral fusion. Attention should be paid to insertion point of vertebral screw close to the adjacent endplate of involved disc in order to avoid the damage of segmental vessels and iliac lumbar vein.

**Figure 5:**
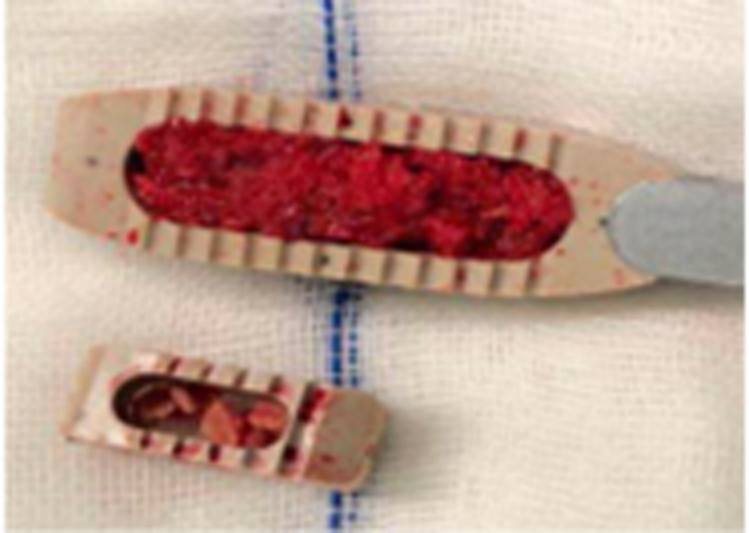
The bigger cage of OLIF has more graft bone and more touch surface than that of MIS-TLIF. OLIF, oblique lumbar interbody fusion; MIS-TLIF, minimally invasive surgery-transforaminal lumbar interbody fusion.

The cage used in OLIF is much bigger than that in MIS-TLIF, which is beneficial for restoration of lumbar anatomy sequence. Postoperative intervertebral space height, lumbar lordotic angle, and operative segmental lordotic angle significantly improved in both groups; there were no significant changes 2 years after operation, and OLIF had significantly more intervertebral space height and operative segmental lordotic angle than MIS-TLIF postoperatively in this study. A bigger cage of OLIF has more touch surface between endplate of vertebra and cage and more graft bone than that of MIS-TLIF (Figure 5), which results in better fusion of OLIF compared with MIS-TLIF. OLIF achieved higher fusion grade than MIS-TLIF in this study, although there was no significant difference between two groups (*p* = 0.194). No subsidence of cage into vertebral body was found in group of OLIF and MIS-TLIF, which was related to protection of cortical endplate during preparation of intervertebral space. In addition, it is very important to place the cage completely parallel with three directions of sagittal, axial, and coronal planes of intervertebral space especially in OLIF; otherwise, the tip of cage may be put into vertebral body through endplate and the subsidence of cage would happen.

**Figure 6:**
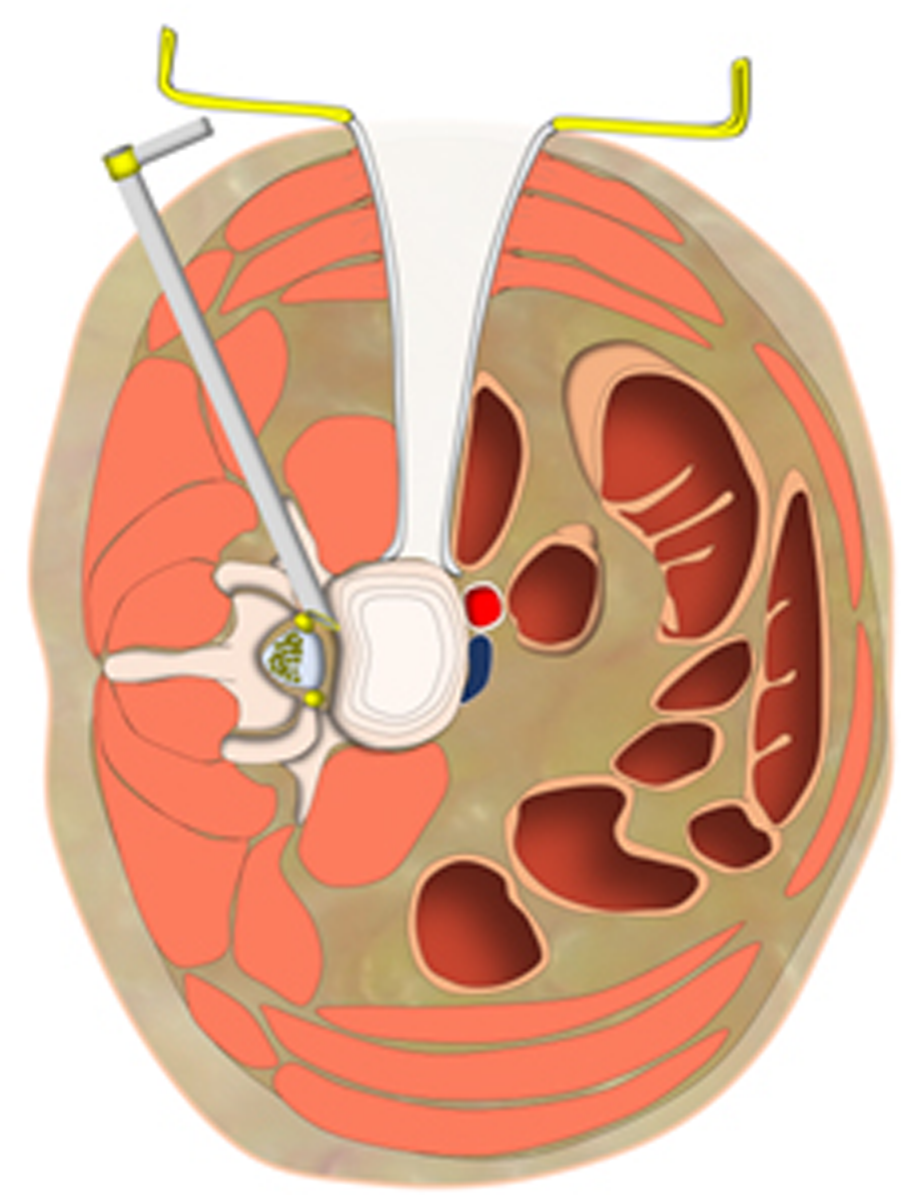
Posterolateral approach of PTES and anterolateral approach of OLIF meet with each other at posterior longitudinal ligament and annulus fibrosus of disc. PTES, percutaneous transforaminal endoscopic surgery; OLIF, oblique lumbar interbody fusion.

We combined PTES under local anesthesia with OLIF and anterolateral screws rod fixation in the same mini-incision for the treatment of single level lumbar spine spondylolisthesis. Posterolateral approach of PTES and anterolateral approach of OLIF meet with each other at posterior longitudinal ligament and *annulus fibrosus* of disc (Figure 6). In PTES, the length of incision was 8.0 mm ± 1.2 mm (Figure 1J) and only ventral bone of articular process was removed, which can be filled into the cage of OLIF. The natural corridor was utilized to place the cage, screws, and rod through the incision of 39.4 mm ± 3.1 mm (Figure 1P) for OLIF and anterolateral screws rod fixation. This combination of two minimally invasive surgeries protects the paraspinal muscles and bone structures as much as possible. MIS-TLIF does not destroy the attachment of paraspinal muscles to bone, supraspinal, and interspinal ligaments, but splits the paraspinal muscles and removes lamina and facet joint. The blood loss of 80 (50–310) ml in MIS-TLIF was significantly more than 30 (15–110) ml in PTES combined with mini-incision OLIF and anterolateral screws rod fixation (*p* < 0.001). The preoperative back VAS score significantly improved postoperatively in both groups (*p* < 0.001), and the back VAS score of group A was statistically lower than that of group B immediately after surgery (*p* < 0.001), which indicates that PTES combined with mini-incision OLIF and anterolateral screws rod fixation has quicker postoperative back pain relief than MIS-TLIF. There was no statistical difference in fluoroscopy frequency (7/5–10 times vs. 7/6–11 times) between mini-incision OLIF and MIS-TLIF, and another fluoroscopy of 6 (5–8) times were needed for PTES besides OLIF in group A, but which had limited influence. Compared with general anesthesia, local anesthesia had little influence on physical status. PTES performed under local anesthesia only needed 49.6 ± 7.8 min and did not prolong the operative duration of general anesthesia for OLIF and anterolateral screws rod fixation in group A, which (74.5 ± 13.5 min vs. 103.9 ± 17.8 min, *p* < 0.001) was significantly less than that for MIS-TLIF in group B. The natural corridor for OLIF and anterolateral screws rod fixation made postoperative drainage fluid little, and when less than 20 ml/24 h the drain tube was removed 2 (1–3) days after surgery and the patients could leave the hospital as soon as possible with the hospital stay of 4 (3–5) days. MIS-TLIF through paraspinal muscle-splitting approaches and open of spinal canal had more drainage fluid, significantly more drain removal time of 4 (3–7) days (*p* < 0.001), and longer hospital stay of 7 (6–10) days (*p* < 0.001).

In MIS-TLIF, dural sac and nerve root should be exposed for neurologic decompression and they must be retracted to insert the cage, sometimes there is the dural tear. In group B, two cases of dural tear occurred, and the lumbodorsal fascia was sutured very tightly to prevent cerebrospinal fluid leakage from surgical incision. There was cerebrospinal fluid leakage from drain in these two patients; the drainage tubes were removed 7 days after surgery when the wound healed and no other abnormal symptoms were found. In group A, two cases had hip flexion pain and weakness possibly related with traction of iliopsoas muscles during OLIF and anterolateral screws rod fixation, which improved during 1 week after surgery. Postoperative radiographs and CT scans showed that the position of cage and screws was good, and no failure of instruments was observed during the 2-year follow-up. No patients had any form of permanent iatrogenic nerve damage and a major complication. All these confirmed the safety of PTES combined with OLIF and anterolateral screws rod fixation for the treatment of lumbar spondylolisthesis, and its complications rate was similar to MIS-TLIF.

There are also some limitations in this study. It is a single-center retrospective study with a relatively small number of patients. This study only includes OLIF25 from L2–5 for the treatment of L2–4 spondylolisthesis because OLIF51 of L5/S1 for L5 spondylolisthesis has different approach and cage. Therefore, we will perform a multicenter prospective controlled study and further study of OLIF for the treatment of L5 spondylolisthesis.

## Conclusion

PTES combined with mini-incision OLIF and anterolateral screws rod fixation has some advantages over MIS-TLIF including smaller aggression, less blood loss, less operative duration under general anesthesia, quicker postoperative back pain relief, better restoration of sagittal lumbar parameter, and better fusion. For both methods, the long-term clinical efficacy and complications rate are comparable. PTES combined with mini-incision OLIF and anterolateral screws rod fixation is a good choice of minimally invasive surgery for lumbar spondylolisthesis, which hardly destroys the paraspinal muscles and bone structures.

## Data Availability

The raw data supporting the conclusions of this article will be made available by the authors, without undue reservation.
